# Forcing of late Pleistocene ice volume by spatially variable summer energy

**DOI:** 10.1038/s41598-018-29916-3

**Published:** 2018-08-01

**Authors:** Kristian Agasøster Haaga, Jo Brendryen, David Diego, Bjarte Hannisdal

**Affiliations:** 1 Department of Earth Science, University of Bergen, P.O. Box 7803, N-5020 Bergen, Norway; 2 The K.G. Jebsen Centre for Deep Sea Research, P.O. Box 7803, N-5020 Bergen, Norway; 3 Bjerknes Centre for Climate Research (BCCR), Allégaten 70, N-5007 Bergen, Norway

## Abstract

Changes in Earth’s orbit set the pace of glacial cycles, but the role of spatial variability in the insolation forcing of global ice volume remains unknown. Here, we leverage the intrinsic dynamical information in empirical records to show that ice volume responded to summer energy at high northern latitudes, as predicted by Milankovitch theory. However, the external forcing of ice volume encompasses insolation signals with a wide range of orbital frequency content, and cannot be fully accounted for by a unique time series. Southern mid-latitude insolation forcing coincides with the position of the subtropical front and the westerlies, which have been implicated in Quaternary climate changes. Dominant forcing modes at northern mid-latitudes are anti-phased with the canonical Milankovitch forcing, consistent with ice volume sensitivity to latitudinal insolation gradients.

## Introduction

Earth’s climate and global ice volume oscillated in tune with changes in orbital geometry during the Quaternary period. Evidence of this relationship is derived from geochemical indicators in marine microfossils found in deep ocean sediment cores. Isotopic ratios in foraminiferal shells (tests) show that Northern Hemisphere ice sheets grew and receded periodically^1^. This periodicity is clearly visible in global sea level reconstructions^2^ and is also mirrored in other climate indices such as global sea surface temperature^3^. Because Earth’s rotational axis and the shape of its orbit vary with similar frequencies as the glacial cycles, the orbital changes have been dubbed a pacemaker of Quaternary glacial-interglacial climate variability^1^. Hypotheses for the origin of the recurrent ice ages include obliquity, precession and combined orbital pacing of deglaciations by various mechanisms^1,4–12^. The extent to which latitudinal insolation acted as a dynamical forcing of late Pleistocene ice volume variability, however, has not been directly quantified.

In the Pleistocene, large paleo-ice sheets were located in the Northern Hemisphere and summer ablation is considered a key factor controlling ice sheet size. Milankovitch theory predicts that if insolation controls continental ice sheet dynamics, then northern latitude summer insolation plays a central role^13^. We test this prediction by quantifying the dynamical response of global ice volume to latitudinal insolation forcing. To achieve this, we target a recent reconstruction of global sea level (GSL)^2^ spanning the past 800,000 years. The advantage of using GSL over stacked benthic isotope records (e.g.^14^) is that it explicitly records global ice volume changes, and that it minimises bias due to temperature-driven fractionation and differences in oxygen isotopes between the Atlantic and Pacific basins^2^.

The GSL time series does not simply record a response to insolation forcing, but contains information on greenhouse gases, ocean circulation, and other processes in the climate system of which ice volume itself is an active component. However, dynamical systems theory shows that one can reconstruct the dynamics of a system of unknown complexity from an observed time series of a single variable^15–17^. Hence, if latitudinally varying insolation was a dynamical influence on changes in ice volume, then information about this insolation forcing should be recoverable from the GSL record of ice volume variations. To test this assertion, we use a model-free time series analysis method, convergent cross mapping (CCM)^18,19^. This method is based on state-space reconstruction from time delay embedding, and measures the extent to which a forcing time series can be predicted from a response time series. CCM can detect causal coupling in non-linear dynamical systems^18^, making the method well suited for studying climate dynamics, where non-linear interactions are ubiquitous. We use CCM to test if GSL can predict latitudinal insolation reconstructions. If GSL significantly predicts the insolation time series at a given latitude, then there is empirical support for local insolation at that latitude contributing to the dynamical forcing of ice volume.

## Results

### Summer energy time series

Ice sheets are most sensitive to insolation during summer melting season. Choosing a meaningful insolation metric is thus crucial for investigating climate system responses to insolation forcing. Summer energy is defined as the sum of daily insolation over days of the year exceeding a specified insolation intensity threshold^20^ and varies with latitude and the choice of the threshold value^21^.

**Figure 1:**
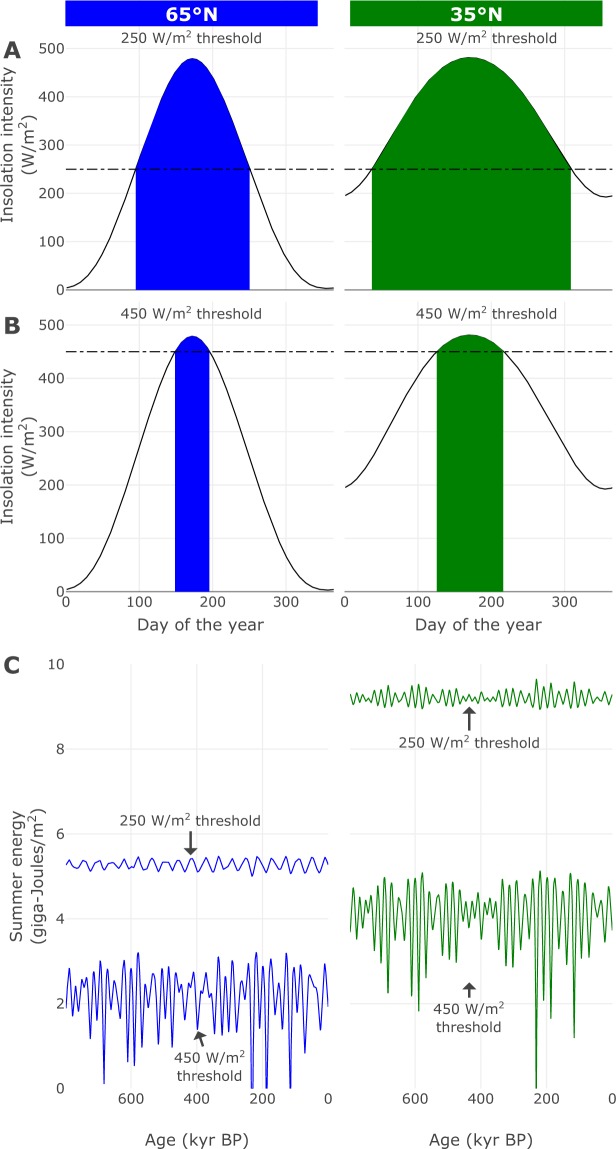
Examples of summer energy reconstructions at different latitudes. (**A**) Yearly insolation intensity (solid lines) at 65°N and 35°N for the current orbital configuration. For low threshold values (stippled line), local summer energy is integrated over large portions of the year (shaded area). (**B**) For higher threshold values, fewer days are integrated, with summer energy representing the local insolation forcing during peak summer. (**C**) Corresponding summer energy forcing time series reconstructed for orbital configurations over the past 800 kyr. Latitudinal summer energy is computed using the code accompanying ref.^20^, which calculates daily insolation following ref.^67^ with orbital parameters from ref.^68^.

We use summer energy time series over all latitudes in 1° increments, generated at threshold values ranging from 0 *Wm*^−2^ to 500 *Wm*^−2^ in 25 *Wm*^−2^ increments. Integrating summer insolation over low thresholds yields time series representing longer summer periods, including the full annual insolation. Higher thresholds, on the other hand, yield time series representing peak summers (Fig. 1). Each summer energy time series corresponds to spatially separate physical forcing scenarios that span different portions of the year, and we seek to detect the dynamical influence of these local processes on global ice volume. We emphasize that we do not use these 1° increment insolation signals to force an ice sheet model. Instead, we use a model-free approach to quantify the dynamical contribution of local insolation at different latitudes on global ice volume.

According to Milankovitch theory, the strongest forcing is expected to occur at latitudes where landmasses were ice-covered during glacial intervals, with weaker or no latitudinal forcing in southern and equatorial parts of the globe. In addition, peak summer is expected to dominate the insolation forcing. Our spatiotemporally explicit approach allows us to test both aspects of the theory without making mechanistic assumptions.

### Significance assessment

We proceed by quantifying the extent to which GSL predicts each latitudinal summer energy time series. To determine whether the integrated insolation forcing at a given latitude is significant, we use the method of surrogate testing (see methods). A necessary condition for statistical detection of a causal response of ice volume to insolation forcing at a given latitude is that the prediction of summer energy time series from GSL is significant beyond their shared frequencies. This condition is tested by a null ensemble of amplitude-adjusted Fourier transform (AAFT) surrogates, which are constructed by randomising the data in a way that retains the autocorrelation function, or, equivalently, the periodogram of the original forcing time series. In order to resolve causal directionality, we compute CCM over a range of negative and positive time lags, and require that CCM skill must be stronger for negative lags (causal) than for positive lags (non causal; see methods). If both the surrogate test and the lag test pass, there is statistical evidence of dynamical forcing of sea level by insolation at that latitude.

### Predicting summer energy from GSL on an orbitally independent age model

**Figure 2:**
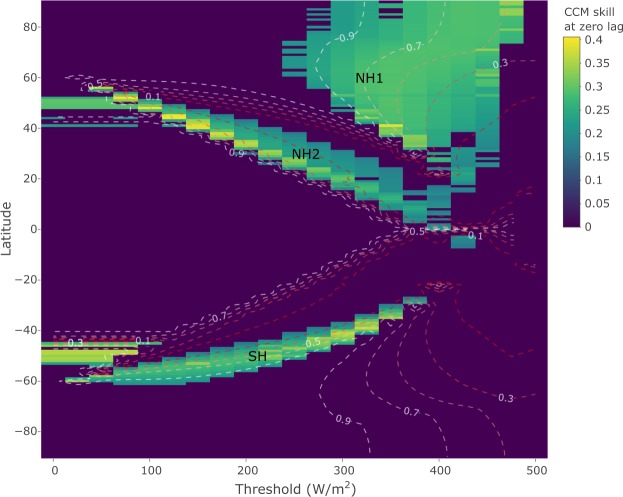
Predicting summer energy from global sea level records for the past 800,000 years. Each cell in the heat map indicates the CCM prediction skill when GSL is used to predict summer energy at that latitude and threshold. Low thresholds represent forcing by insolation over large portions of the year, whereas high thresholds represent peak summer insolation forcing. Non-zero skill implies that GSL contains information about summer energy beyond noise and shared frequencies, which, in the context of CCM, is interpreted as dynamical forcing of ice volume by summer energy at that latitude. The magnitude of CCM skill indicates the relative strength of summer energy forcing. Skill is set to zero for latitude-threshold specifications where the lagged causality test fails and/or the null hypotheses cannot be rejected at the 0.01 level (see methods). Contour lines indicate the fraction of obliquity (1/41 ± 1/150 kyr) to precession (1/21 ± 1/150 kyr) frequency band power for the corresponding summer energy time series, from obliquity dominated (dark red lines) to precession dominated (white lines).

CCM analysis yields three connected regions of positive prediction skill in latitude-threshold space (Fig. 2). These three clusters represent insolation forcing occurring during different portions of the year in distinct latitudinal bands.

In the latitudinal zone at 50–90°N, GSL predicts summer energy time series for a wide range of threshold values, primarily above 250 *Wm*^−2^ (cluster NH1 in Fig. 2). Prediction strengths in this latitudinal band are bi-modally distributed south and north of 70°N. North of 70°N, prediction is strongest when summer energy is defined by the 400–450 *Wm*^−2^ thresholds. South of 70°N, prediction strength peaks at the 300–350 *Wm*^−2^ thresholds. This prediction pattern coincides with the relative dominance of orbital frequencies in the forcing time series: prediction skill is strongest for time series with roughly equal contributions of obliquity and precession (contour lines in Fig. 2). From the maximum prediction strength at around 50/50 obliquity/precession, prediction skills decrease as the summer energy time series get more precession or obliquity dominated; this corresponds to insolation integrated over shorter and longer summer time windows, respectively.

**Figure 3:**
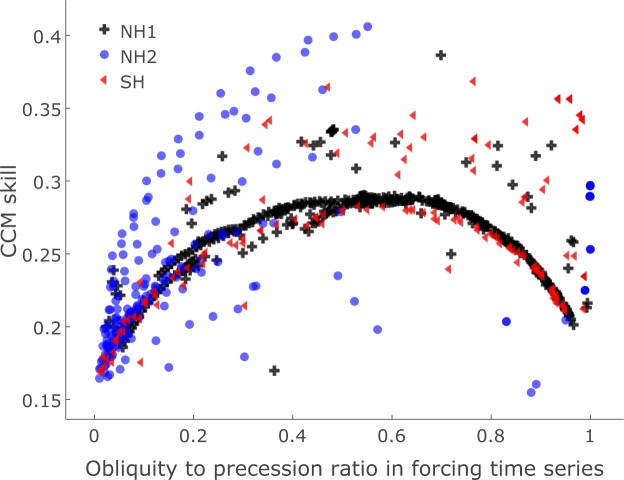
Ratio of obliquity band (1/41 ± 1/150 kyr) to precession band (1/21 ± 1/150 kyr) variance in summer energy time series versus strength of coupling to ice volume indicated by CCM skill. Results are grouped according to the clusters in Fig. 2.

Further, we detect significant prediction of summer energy by GSL in two continuous clusters in latitude-threshold space, one in each hemisphere (clusters NH2 and SH in Fig. 2), where GSL predicts summer energy, primarily for time series corresponding to thresholds of 50–400 *Wm*^−2^. The overall pattern for both clusters is that prediction is successful at lower latitudes for high threshold values, transitioning to increasingly higher latitudes as the threshold decreases. However, these clusters are hemispherically asymmetric. NH2 spans the latitudinal zone from 55°N to around the equator, while SH covers 30–65°S. GSL predicts time series with a wide range of obliquity-to-precession ratios for both clusters, but there are some cross-hemispheric differences (Fig. 3). SH corresponds to time series with frequency power distributed over the entire range of obliquity/precession ratios, while time series are more precession dominated for NH2. Annual integrated insolation at low threshold specifications (0–75 *Wm*^−2^) is also predicted by GSL. At these thresholds, successful prediction occurs in both hemispheres 45–55° (included in NH2 and SH).

**Figure 4:**
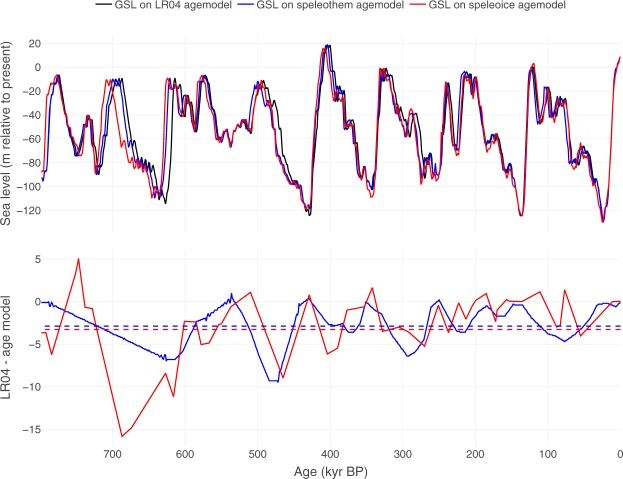
Age models used in this study. Upper panel: the black line shows GSL on the original age model^2^, which is aligned with the LR04 stack^14^. The blue line shows GSL on the age model (‘speleothem’) constructed following the approach of ref.^22^ using band-pass filtering (see methods). The red line shows GSL on an orbitally independent age model (‘speleoice’) constructed by tuning a composite of North Atlantic SST proxy records to the speleothem record (see methods). Lower panel: age offsets between the original LR04-based GSL chronology and the two alternative age models. Dashed lines show the average offsets.

**Figure 5:**
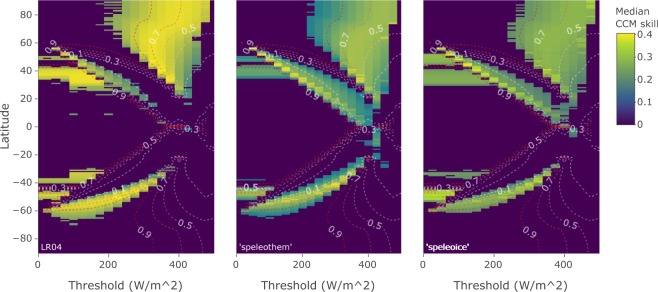
Comparison of non-lagged CCM results for the different age models considered in this study (see Fig. 4). Overall, CCM skills are higher for the LR04 age model, possibly reflecting stronger coupling induced by the tuning of the LR04 age model to the orbital forcing. All results discussed in the main text are based on the fully orbitally independent ‘speleoice’ age model.

We have verified our results on three different age models for the GSL record. The original GSL age model is based on the LR04 stack, whose age model is tuned to an ice sheet model forced by insolation. This approach may introduce circularity between the orbital forcing and the putative ice volume response. Therefore, we have constructed two alternative age models for the sea level stack (Fig. 4; see methods). One is based on tuning by aligning bandpass-filtered sea level and speleothem records^22^, and one utilises the connection between North Atlantic sea surface temperatures and Asian monsoon intensity, linking North Atlantic benthic *δ*18O records with U-Th-dated Chinese speleothem records^23,24^. Both alternative age models are based on U-Th and ice core data, but the latter uses a tuning independent of both ice volume and orbital parameters. The two alternative models give overall similar non-lagged CCM results as the LR04 age model when predicting summer energy from GSL (Fig. 5). To avoid circularity, we here present results based on the age model that is independent of both sea level and orbital tuning.

## Discussion

Our model-free approach, which makes no *a priori* assumptions about the coupling between ice volume and insolation, neither through explicit mechanisms nor through age model construction, provides strong evidence of Milankovitch type forcing of ice volume. Northern Hemisphere ice sheets dominated the global ice volume signal during the past 800,000 years. The latitudinal zone from 50–80°N corresponds to the known range of land-based Northern Hemisphere ice sheets during the late Pleistocene, which reached as far south as 40°N during glacial maxima^25^. The successful prediction of summer energy by GSL in the NH1 cluster (Fig. 2) thus provides a data-driven confirmation that Northern Hemisphere summer energy acted as a dynamical forcing of Northern Hemisphere ice sheets.

We re-iterate that CCM does not make assumptions about properties or mechanistic behaviors of the ice sheets, or their interaction with other climate system components. Our approach only targets the intrinsic dynamical information in the observed GSL record, and estimates the strength of dynamical coupling over the entire 800 kyr interval covered by the GSL reconstruction. This way of looking at dynamical causality is fundamentally different from an event-based view^26^, in that an underlying dynamical coupling between insolation and the climate system might exist even if the relationship between components varies through time (e.g. lead-lag relationships between glacial terminations and insolation peaks). Thus, the detected couplings in our study capture not only direct, linear responses of ice sheets to specific insolation peaks, but also nonlinear, lagged effects which include modulation by other climatic factors such as greenhouse gases.

Causal pathways from local insolation to global ice volume vary with latitude. Different forcing scenarios overlap in their relative ratio of obliquity to precession variability (Figs 2 and 3) and might show strong covariance, but unique latitude-threshold combinations correspond to physically distinct causal chains leading from local insolation to ice volume variations. An example of this distinction is Antarctic climate, which, due to its phase coherence with selected Northern Hemisphere insolation time series, has been interpreted to be controlled by northern insolation^27^. However, Antarctic climate variability can also be explained by a local response to the duration of the Antarctic summer^28^. Both local and remote forcings might thus influence a given geographical region. Because of the uncertainty inherent in ice sheet reconstructions, the boundary separating regions of direct insolation influence from regions of indirect influence cannot be precisely resolved. The NH1 cluster represents mostly direct insolation forcing effects, but likely also some indirect effects for the southernmost forcing signals. Similarly, the northernmost forcing signals in NH2 may represent the direct effect of annual insolation on ice sheets at these latitudes.

**Figure 6:**
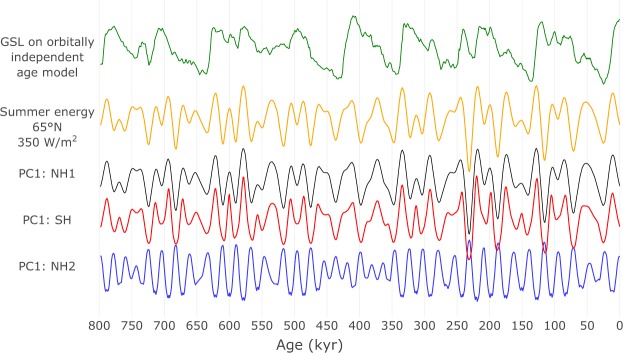
Comparing dominant latitudinal summer energy modes to the canonical Milankovitch forcing signal. The first principal component (PC1) time series of the three significant forcing clusters in Fig. 2 explain 94% (NH1), 77% (NH2), and 82% (SH) of the variance in each cluster. The conventional Milankovitch forcing is represented by summer energy at 65°N for the 350 *Wm*^−2^ threshold. For reference, the upper time series shows the sea level record (GSL) used to predict insolation curves (for details on the orbitally independent age model, see Fig. 4).

Our analyses indicate that Northern Hemisphere ice sheets respond to local summer insolation, but that insolation in the SH cluster (Fig. 2) also contributes to ice volume variations. Although the dominant modes of summer energy forcing in the NH1 and SH clusters are redundant (Fig. 6; black and red lines), individual forcing time series are highly variable (Fig. 3). Our interpretation is that there are distinct physical processes occurring during different times of the year in different geographical regions, each having a unique causal pathway to ice volume, that may have worked in tandem to produce the observed global ice volume variations. Covariance between different local summer energy forcing time series might be strong, but local climate necessarily responds to the duration of summer and the magnitude of the integrated insolation intensity at that location, the effect of which may be magnified or suppressed by other climatic processes.

There are several climatic processes operating at southern mid-latitudes that might be significantly influenced by local insolation. For example, the Patagonian ice sheet resides at these latitudes, and it has been hypothesized that Patagonian ice sheet dynamics affect the flux of dust over the Southern Ocean and Antarctica^29^. Variation in the dust supply to surface waters drives natural iron fertilisation and regulates the intensity of the biological pump^30^. This process could influence ocean stratification and venting of CO_2_ from the deep oceans, resulting in global climatic impacts. We also note that the 40–60°S latitudinal band coincides with the position of the oceanic subtropical and sub-Antarctic front systems and the mid-latitude westerlies^31^. It has been proposed that the position of the subtropical front relative to the southern tip of Africa modulates glacial climate through regulation of heat and salt exchange between the Indian and Atlantic oceans by the Agulhas leakage, affecting the strength of the Atlantic meridional overturning circulation^31–33^. In addition, the position of the westerlies and Southern Ocean sea ice dynamics may regulate venting of deep water and the release of stored CO_2_ to the atmosphere, which in turn affects ice sheets in the Northern Hemisphere^34^.

Our analysis shows no statistically significant effect of southern high latitude summer energy on global sea level. The response to direct insolation forcing at high southern latitudes may be too small in comparison to that of northern hemisphere ice sheets for it to be detectable beyond our chosen null hypothesis. This result may be understood in terms of the difference between the Arctic and Antarctic ice sheets. Because the Antarctic ice sheet mostly loses mass along the continent edges^28^, it is less affected by albedo and elevation feedbacks^35^. Its influence on global sea level on the time scales considered here could thus be negligible compared to the Northern Hemisphere ice sheets, which show much larger variability over the same time span.

In addition to the direct effect of local northern insolation, Northern Hemisphere ice sheets were likely also influenced by local and global feedbacks involving basal sliding^36^, greenhouse gases^34^, changes in vegetation^37^, dust^38^ and glacial isostasy^39^. The explicit role of different mechanisms in the insolation-climate link, however, cannot be resolved with the global data sets used here. For regions in the Northern Hemisphere south of the maximal extent of the late Pleistocene ice sheets, the influence of local summer energy forcing on ice volume must necessarily be indirect. Overall, insolation forcing in the NH2 cluster is overall more precession dominated compared to further north in NH1 (Figs 2, 3 and 6). The sensitivity of local climatic processes to local insolation forcing at these latitudes was likely different from regions hosting large Northern Hemisphere ice sheets, where the detected insolation forcing is mostly restricted to the summer half-year. In the NH2 cluster, the dominant forcing mode is generally in anti-phase and inversely correlated with the direct Milankovitch type summer forcing of more northern latitudes (Fig. 6; blue line). This inverse relationship is consistent with a role of differential heating due to meridional insolation gradients in the Northern Hemisphere, which may regulate atmospheric fluxes of moisture and heat^40,41^.

From a linear perspective, we can distil the variance of clusters of summer energy time series in latitude-threshold space using the eigenvector of the covariance matrix of each cluster with the largest corresponding eigenvalue (Fig. 6). For both NH1 and SH clusters, their first principal component time series nearly perfectly covary with 65°N summer energy at the 350 *Wm*^−2^ threshold, which closely matches the caloric summer half-year insolation at 65°N that was considered by Milankovitch^13^. Using 65°N summer energy at 350 *Wm*^−2^ as a first approximation of regional insolation forcing of global ice volume on these time scales is thus supported by intrinsic dynamical evidence in the GSL record on an orbitally independent age model. In contrast, the first principal component time series for NH2 is more precession dominated and generally in antiphase with the NH1 and SH principal components. Hence, the dynamics of the summer energy time series in the NH2 cluster represents regional insolation forcing of global ice volume that is not captured by the canonical Milankovitch forcing. We emphasize that although the dominant insolation forcing modes for the NH1 and SH clusters are similar to the canonical Milankovitch forcing (Fig. 6), significant predictability occurs for a range of time series with very different characteristics within each cluster. The nature of our causality test ensures that any inferred dynamical coupling between global ice volume and summer energy on these time scales cannot be accounted for solely by the dominant modes of forcing or by orbital frequency content alone.

Both obliquity and precession components feature prominently in the dynamics of global ice volume. Our analysis shows that there is no clear answer to the question of which orbital parameter plays a greater overall dynamical role as a forcing of global ice volume (Figs 2 and 3). The spread of obliquity-to-precession frequency content in significantly predicted summer energy time series arises naturally as a consequence of spatiotemporal heterogeneity in the insolation forcing.

## Methods

### Convergent cross-mapping (CCM) analyses

If two variables belong to the same dynamical system, then there is a 1:1 mapping between the reconstructed state spaces of both variables^16^. CCM estimates to what extent such a mapping exists, using the amount of information from the “driving” variable that is encoded in the “response” variable, and vice versa^18^. To resolve causal directionality, we employed time-lagged analyses using the *rEDM* implementation^42^ of CCM. Rather than inferring causality whenever the optimal lag is negative^19,43,44^, we used a more stringent criterion to reduce the likelihood of false positives: the total significant CCM skill (*ρ*_*ccm*_) of negative lags (past affects future) had to exceed that of positive lags (future affects past):

$${\rho }_{ccm}^{causal}=\sum _{i=min(lag)}^{-1}\frac{{\rho }_{ccm}^{i}}{{n}_{lags}}-\sum _{i=1}^{max(lag)}\frac{{\rho }_{ccm}^{i}}{{n}_{lags}}$$

where results for zero lag were excluded to avoid bias in either direction. A directional causal forcing was detected if ${\rho }_{ccm}^{causal} > 0$. In contrast, ${\rho }_{ccm}^{causal}\le 0$ implied that there was no detectable causal effect. We chose this conservative approach to limit the likelihood of false positives; we did not infer strict causal delays, which might be biased^45^, but limited our interpretation to causal directionality. Our lagged causality test (Fig. 2) used a maximum lag of 12 kyr.

### Choice of embedding dimensions

CCM estimates dynamical coupling using a time-delay reconstruction^16,17,46^ of the dynamics. We took the minimal embedding dimension (*E*_*min*_) as the integer dimension strictly larger than twice the box-counting dimension^17^ of the reconstructed attractor, and required the false nearest neighbour (FNN) rate to be less than 0.01. We used the *tseriesChaos*^47^ and *fractaldim*^48^ R packages to estimate the minimal FNN (${E}_{min}^{FNN < 0.01}$) and box counting dimensions (${E}_{min}^{box}$). To ensure numerical stability when estimating the box-counting dimension, summer energy time series with >2% zero values were excluded from the analyses. Optimal embedding parameters *E*_*opt*_ were then selected by maximizing self-prediction using simplex projection^49^ over integer dimensions $\{max\{{E}_{min}^{box},{E}_{min}^{FNN < 0.01}\},\ldots ,10\}$ with embedding lag 1, setting 10 as the maximum embedding dimension to limit computational cost. Optimal embedding parameters were estimated separately for each pair of GSL-insolation time series. For each cross mapping, we constructed embeddings using the optimal embedding dimension for the target variable (the presumed driver). Leave-*k*-out cross validation with an exclusion radius of 30 kyr was used on prediction libraries to limit autocorrelation bias.

### Statistical acceptance criteria

CCM requires that the correlation between predicted and observed values increases with increasing library size^18^. We determined convergence by regression of *ρ*_*ccm*_ on 20 different library sizes (*L*) in the range $(E+tau+max(lag,1),\mathrm{...},L)$ distributed among the smallest possible and largest possible library sizes. Analyses were labelled convergent if the value of the constant *q* in the expression $\rho ={\rho }_{max}-{\rho }_{0}\cdot {e}^{-q(L-{L}_{0})}$ was positive; here, *ρ* is the median CCM predictive skill and *L* is the library size. The value of *q* was found through linear regression of the logarithmic transformation of the same equation, or $q=[ln({\rho }_{0})-ln({\rho }_{max}-\rho )]/(L-{L}_{0})$. In addition, to be convergent, we tested whether CCM skills were higher at the largest library sizes compared to the lowest library sizes by the means of a Wilcoxon rank sum test, which had to reject the null at the 0.01 level. Non-convergent analyses were discarded from the calculation of ${\rho }_{ccm}^{causal}$.

The upper limit of the CCM skill for a given analysis is determined by the coupling strength between the variables, but also by process noise^18^. Therefore, to claim significant forcing, we establish a distribution in the form of an ensemble of amplitude-adjusted Fourier transform (AAFT) surrogate time series^50^. These surrogates are randomized realizations of the original time series that preserve both the histogram and the frequency power spectrum (i.e. autocorrelation) of the original insolation forcing time series. Rejecting the null hypothesis thus implies that the dynamical coupling between GSL and the insolation cannot be fully accounted for neither by noise properties nor by shared frequencies.

Rejection of the null hypothesis for each driver-response pair involved passing a one-sided rank-order test^51^ where the *ρ*_*ccm*_ of the data had to exceed the 99^*th*^ percentile *ρ*_*ccm*_ of the surrogate ensemble. We used 400 surrogates, and verified the results at selected threshold-latitude configurations using 1,000 surrogates.

### Orbitally independent chronology

We explored three different chronologies for the GSL record (Fig. 4). The original age model for the GSL record^2^ is aligned with the LR04 stack^14^, which is tuned to an orbitally forced ice sheet model. We constructed an alternative age model following the approach of ref.^22^, wherein a filtered GSL (band-pass filtered using a 22 ka Gaussian filter) was tuned to an equivalently filtered composite *δ*^18^O record from U/Th dated Chinese speleothems^24^. GSL was then aligned with the speleothem record by tiepoints determined from peaks and troughs in the band-pass filtered versions. The speleothem record goes back to 640 kyr; age control for the older parts of the GSL record was obtained by linear interpolation to a tie point on the LR04 stack at 787 kyr, close to the Brunhes-Matuyama boundary. In an effort to obtain an orbitally independent chronology, we constructed another GSL age model by tuning a North Atlantic SST composite proxy record to the speleothem record. The GSL record was then matched to the benthic *δ*^18^O records of the respective North Atlantic archives. This approach utilizes the close connection between millennial-scale North Atlantic climate and the intensity of the Asian Monsoon documented in several studies^52–54^. In the interval from 0 to 332 kyr we used the GICC05/NALPSpeleo chronology from ODP site 983^23^ with a benthic *δ*^18^O record from ref.^55^. In the interval from 332 to 553 kyr we used the SST record from IODP site U1313^56^ and the corresponding benthic *δ*^18^O record^56,57^. In the interval older than 553 kyr, we used the abundance of the polar planktonic foraminifer *Neogloboquadrina pachyderma* sinistral and the benthic *δ*^18^O records from ODP site 980^58^. Age control beyond the reach of the speleothem composite was obtained by matching the ODP 980 data to the Epica Dome C methane record^59^ placed on a modified AICC2012 gas age chronology. The AICC2012^60^ was modified by matching the methane record to the composite speleothem record from 554 to 627 kyr, and to two tie points at 783 and 792 kyr determined by Ar/Ar dated tephras and a *δ*^18^O record from an Italian lacustrine sediment sequence^61^, utilizing the close relationship between the atmospheric methane concentration, North Atlantic climate and Asian Monsoon intensity (e.g.^62,63^). Age models were constructed with *Oxcal 4.3*^64^, using the *P* sequence^65^ and variable *k*^66^ options.

### Code

Code to reproduce all analyses and figures is available at https://github.com/kahaaga/Haaga_et_al_insolation.
